# Plasma, Urinary, Erythrocyte and Platelet Zinc Concentrations in Soccer Players

**DOI:** 10.3390/nu16162789

**Published:** 2024-08-21

**Authors:** Víctor Toro-Román, Jesús Siquier-Coll, Fco. Javier Grijota Pérez, Marcos Maynar-Mariño, Ignacio Bartolomé-Sánchez, María C. Robles-Gil

**Affiliations:** 1 Research Group in Technology Applied to High Performance and Health, Department of Health Sciences, Universitat Pompeu Fabra, TecnoCampus, 08302 Mataro, Spain; vtoro@tecnocampus.cat; 2 IM-PEPH (Improving Physical Education, Performance, and Health), Department of Communication and Education, University of Loyola Andalucía, 41704 Sevilla, Spain; 3 Department of Physiology, Sport Science Faculty, University of Extremadura, 10003 Caceres, Spain; mmaynar@unex.es (M.M.-M.); mcroblesgil@unex.es (M.C.R.-G.); 4 Faculty of Health Sciences, Isabel I University, 09003 Burgos, Spain; 5 Faculty of Education, University Pontificia of Salamanca, 37007 Salamanca, Spain; ibartomesa@upsa.es

**Keywords:** trace minerals elements, sex differences, biological matrices, longitudinal study

## Abstract

Essential trace minerals are vital for general human health and athletic performance. Zinc (Zn) plays critical roles in several biochemical processes in exercise physiology, especially during intense physical exercise. This research aimed to analyze erythrocyte, platelet, plasma and urine Zn concentrations among men’s and female soccer players over a sports season. A total of 22 male soccer players (20.61 ± 2.66 years; 71.50 ± 5.93 kg) and 24 female soccer players (23.37 ± 3.95 years; 59.58 ± 7.17 kg) participated in this longitudinal quasi-experimental study. Three assessments were carried out over the season: 1st evaluation: first week of training (August); 2nd assessment: middle of the season, between the end of the first and second round (January) and 3rd assessment: last week of training (May/June). In all evaluations extracellular (plasma and urine) and intracellular (erythrocytes and platelets) Zn concentrations were determined, as well as physical fitness and several blood parameters. Inductively coupled plasma mass spectrometry was used to measure Zn concentrations. Plasma and urinary concentrations were higher among male soccer players (*p* < 0.05) while erythrocyte and platelet Zn concentrations were higher in the female soccer players (*p* < 0.05). Additionally, variations in urinary and platelet Zn concentrations were observed over the season. The differences could be related to muscle mass, muscle damage or the specific sport’s physical demands.

## 1. Introduction

Zinc is found in various tissues, organs and secretions, being one of the most important trace minerals. Iron (Fe) is the only element that is higher in abundance than zinc [1]. About 95% of Zn is located inside the cells. Zn is present in all cell organelles, with 60–80% located in the cytosol and the remainder bound to cellular membranes [2].

Zn is involved in different actions of the human body during intense physical exercise and is related to muscular strength and endurance [3]. In addition, Zn acts as an antioxidant superoxide dismutase (SOD) together with Cu (Copper) [4]. At the same time, Zn provides structural stability and is involved in enzymatic activities of metalloenzymes, such as lactate dehydrogenase and carbonic anhydrase [5]. The latter is one of the most abundant enzymes in the body, being essential for maintaining the acid–base balance of body fluids [6]. Thus, this mineral plays an important role in high-intensity sports such as soccer.

Long-term physical training produces different adaptations that could influence Zn redistribution [7]. This redistribution seems to depend on the type of physical exercise, training status, exercise duration and environmental temperature [8]. Soccer is a vigorous sport with great physical demands over the season [9], generating a high level of free radicals [10]; therefore the role of Zn is essential in this sport. However, Zn levels can vary over the season for different reasons. High temperatures are an element that can affect Zn levels due to its excretion through sweat, and an increased excretion of this element has been observed in urine, even in acclimated subjects [11]. In addition, soccer is an intense physical sport with a tight competition schedule, which produces higher reactive oxygen species caused by training [12]. The resulting high mitochondrial oxygen consumption and electron transport flux results in oxidative stress leading to the generation of free radicals and, as a consequence, possible lipid peroxidation [13]. Moreover, high impacts result in a greater level of muscle damage, triggering the release of Zn by muscle cells [8]. A recent study has found a connection between creatine kinase (CK) and Zn [14]. There is a high incidence of injuries in soccer [15]. In this respect, it should be noted that these impacts trigger a release of erythrocyte Zn into the plasma. This could be the reason why several studies report elevated plasma/serum Zn in top-level athletes [16,17]. Recent research has shown that athletes who engage in regular physical training have increased zinc levels in their plasma and decreased levels in their erythrocytes [17]. According to these findings, it is important to assess trace minerals not just in extracellular compartments, since the plasma/serum zinc concentration is a limited marker for determining zinc status in humans.

The current literature usually evaluates the status of this mineral in men, and there is a lack of knowledge about the status of Zn in women athletes. It has been observed that Zn concentrations are higher in men than in women before and after a maximal incremental test [18]. In addition, it is known that Zn loss is greater in men than in women under both hyperthermic and normothermic conditions [19]. These differences are mainly explained by a greater muscle mass in men than in women, since most Zn is found in skeletal muscle [1]. Strength outputs, and thus muscle damage, are greater in men during competition, so the release of Zn into plasma is higher [20]. However, the studies that have been carried out evaluate Zn in a single matrix or at a single moment of the season. It seems necessary to establish the behavior of this mineral over the course of a season in different matrices as mentioned above. A recent study reported values of the effect of resistance training in marines [21]. Nevertheless, the kinetics of this mineral may vary depending on the type of exercise. Soccer is a mixed-mode sport. Another factor that can modify Zn kinetics are injuries, as these could cause hemolysis. In addition, it should be noted that the menstrual cycle in women can also alter the homeostasis of Zn over a season, so it would be interesting to study this element in different matrices in women and observe the differences between sexes to know the possible requirements. Therefore, this study aimed to observe Zn concentrations at different moments of the sports season in men’s and female soccer players in the following biological matrices: erythrocytes, platelets, plasma and urine. Based on previous studies [14,17,22], it could be hypothesized that there would be differences between sexes, depending on the matrix analyzed, as well as over the season due to the physical demands of the sport.

## 2. Materials and Methods

### 2.1. Study Design

The present observational research is based on a longitudinal–quasi-experimental design. Three evaluations were carried out over a regular season on two senior soccer teams in the city of Cáceres: CD Diocesano (men) and CP Cacereño (women). Over the study, longitudinal comparisons were made between the players, as well as between the two soccer teams in order to observe possible differences between sexes. The evaluations were carried out at the following moments of the regular sports season: 1st evaluation: first week of training (August); 2nd assessment: middle of the season, between the end of the first and second round (January) and 3rd assessment: last week of training for both teams, once the season ended (May/June).

All assessments were carried out in the Faculty of Sports Sciences at the University of Extremadura. Evaluations were scheduled in the morning (starting blood draws at 8:00 a.m. and at 9:30 a.m. the physical tests) on the same week each month, following a consistent order to minimize the effects of circadian rhythms. Furthermore, the assessments were carried out under similar atmospheric conditions (18–25 °C and 45–55% relative humidity) as they were all conducted at the Faculty of Sport Sciences, where environmental parameters were monitored.

### 2.2. Participants

The study included 46 soccer players who were fully informed of the study purpose and provided written consent before participating. The protocol was reviewed and approved by the Biomedical Ethics Committee of the University of Extremadura (Cáceres, Spain) (code 135/2020) following the guidelines of the Helsinki ethical declaration, updated at the World Medical Assembly of Fortaleza (2013) for research with human beings. Two days prior to the assessments, the training load of both teams was reduced so that the participants could perform the different assessments with as little fatigue as possible.

The participants were divided according to sex and the team they belonged to: male soccer players (n = 22; age = 21.15 ± 2.45 years; height = 1.77 ± 0.06 m; weight = 72.35 ± 5.87 kg; experience = 15.20 ± 3.05 years) and female soccer players (n = 24; age = 24.18 ± 3.98 years; height = 1.66 ± 0.07 m; weight = 60.42 ± 7.38 kg; experience = 14.22 ± 4.88 years). The men were from a fifth-division Spanish soccer team, and the women were from a second-division Spanish team. All the participants trained and played league matches in the same city.

In order to participate in the study, individuals had to meet several inclusion criteria. These included: (i) residing in the same city; (ii) not suffering from any medical conditions; (iii) avoiding medications or supplements containing MTE for at least one month prior to participation and during the study period itself; (iv) completely abstaining from smoking or drug use; (v) having five years of competitive football playing experience; (vi) maintaining consistent nutritional and physical activity habits throughout the experiment; and (vii) not spending more than 30 days away from team training. In addition, female participants were required (viii) to have regular menstrual cycles for six months prior to the start of their participation in this research (and to continue this pattern throughout the study); (ix) not to experience complications related to either menstrual problems or cycle-related issues; and (x) not to have used contraception.

The team coaches provided information on player details, training plans, matches, and injuries, with player consent. Internal training load data were not included due to the different methodologies used by each coaching staff (Table 1).

### 2.3. Menstrual Cycle

The researchers administered an online questionnaire to identify the menstrual cycles of the female soccer players [23]. The participants could obtain help from a researcher while answering the questions. The questionnaire covered aspects like cycle length, bleeding duration and type, age of first menstruation, menstrual regularity and any associated pain or symptoms. Understanding the menstrual cycles was crucial because some studies suggest fluctuations in certain trace mineral concentrations throughout the cycle [24,25]. Consequently, all evaluations, whenever possible, were conducted during the same menstrual phase. Additionally, as detailed later (Table 2), the researchers measured the female hormones progesterone and estradiol-17.

### 2.4. Anthropometrics and Body Composition

Following guidelines by Porta et al. [26], the participants were in a fasting condition and wore minimal clothing during body measurements. The researchers measured the following: height using a wall-mounted stadiometer, weight with an electronic scale and skinfolds at six locations on the participants’ right side (abdominal, suprascapular, subscapular, tricipital, thigh, and calf) with a specialized caliper. All measurements were taken three times with the average value used for analysis.

### 2.5. Maximal Incremental Exercise Test

The maximal incremental test was performed on a treadmill (Ergofit Trac Alpin 4000, Kübler Sport GmbH, Backnang, Germany), equipped with a gas analyzer (Geratherm Respiratory GMBH, Ergostik, Ref 40.400, Corp., Bad Kissingen, Germany) and a Polar heart rate monitor (Polar^®^ H10, Polar Electro, Kempele, Finland). The protocol consisted of running in 1 min stages until exhaustion. The test started at 7 km/h and increased by 1 km/h every minute with a stable gradient of 1%. Prior to the test, a 15 min warm-up on the treadmill was performed where participants ran at 6 km/h. All tests were performed after a free breakfast.

### 2.6. Nutritional Assessment

Similar to the method used by Toro-Román et al. [14], the participants completed a food questionnaire to determine their intake of macronutrients and Zn. Nutritional data collection was carried out using an Excel file. The file was sent individually to each participant. In these files participants had a complete list of foods [27], and they had to indicate the number of meals eaten each day over the 3-day evaluation. Additionally, in each meal, participants selected in the Excel database the specific food as well as the quantities/portions consumed.

Once the Excel questionaries was obtained researchers converted these food entries into estimated numerical values using stablished and validated references [27]. When the specific amounts of foods were obtained, it was introduced in a database, obtained the stablished amounts of Zn in each food consumed [27].

### 2.7. Sample Collection

Prior to the evaluations, trainers for each team were provided containers and tubes as urine collection kits which they distributed among their players. A guideline was given for the participants to collect their initial morning urine samples between 8:00 and 8:30 a.m. and deliver them to the blood extraction station. The collected urine was stored in designated 100 mL containers before being transposed into smaller storage vessels of about 9 mL by each player. Afterwards, these small tubes containing frozen samples were preserved at a temperature of −80 °C until lab analysis could be performed on them later.

Blood samples were collected after overnight fasting. A total of 12 mL of blood was drawn using a syringe and needle. Two milliliters were placed in tubes with a clotting agent to analyze blood cell parameters (using a specific machine) and female hormones (using an ELISA test with a spectrophotometer).

To measure Zn levels, the remaining 8 mL of blood was utilized. From this amount, four milliliters underwent extraction in tubes with sodium citrate as a blood thinner. To isolate plasma or the liquid portion of blood, one tube was subjected to centrifugation or high-speed spinning which caused platelets or cell fragments to settle on top and be collected into another tube that likewise went through a second round of centrifugation, resulting in platelet-rich plasma being separated into smaller vials and then frozen at −80 °C storage temperature. The rest of the bloodstream also had red cells eliminated via centrifugation before washing and preparation for cryopreservation, similarly at −80 °C degrees Celsius coldness level for future use/storage purposes.

### 2.8. Zn Determination

The methods used to extract Zn from different blood components were similar to established methods used in previous research [15].

A special technique called inductively coupled plasma mass spectrometry (ICP-MS) was used to measure Zn levels. This technique has been shown to be reliable, with calibration checks for an element called indium showing a strong correlation (greater than 0.985) and consistent results (coefficients of variation less than 5%).

For plasma and urinary samples, the reagents used were 69% nitric acid (TraceSELECT™, Fluka™, Madrid, Spain) and ultrapure water obtained from a Milli-Q system (Millipore^®^, Burlington, MA, USA). A rhodium dilution of 400 µgL^−1^ was used as the internal standard and continuously fed into the apparatus with the aid of the three-channel peristaltic pump. From the 0.20 mL of samples, a volume of 5 mL was made up with a 1% nitric acid solution prepared from a commercial one of 69% (TraceSELECT™, Fluka™, Madrid, Spain). The equipment was calibrated with several standards prepared from commercial multi-elemental dilutions of certified standards.

For erythrocyte and platelet samples, the reagents used in method development and sample analysis were nitric acid (69%), hydrogen peroxide (TraceSELECT™, Fluka™, Madrid, Spain) and ultrapure water obtained from a Milli-Q system manufactured by Millipore (USA). A 400 µgL^−1^ solution of yttrium and rhodium was used as the internal standard. 

Samples were weighed on precision scales and transferred to glass tubes for microwave digestion, and 3.5 mL of a 3:1 mixture of 69% nitric acid (TraceSELECT™, Fluka™, Madrid, Spain) and hydrogen peroxide (TraceSELECT™, Fluka™, Madrid, Spain) were added. The samples were digested in a Milestone Ultrawave microwave (Sorisole, Italy), and once digested were diluted to 25 mL with MilliQ water. The detection and quantification limits of Zn in the different matrices throughout the investigation are shown in the following Table 3.

### 2.9. Statistical Analysis

The data collected were analyzed using IBM SPSS Statistics 25.0 software (IBM Corp., Armonk, NY, USA), and the outcome is shown as mean values along with a measure of variation (standard deviation and 95% confidence interval for Zn). First, the researchers checked if the data followed a normal distribution using a statistical test called the Shapiro–Wilk test. For normally distributed data (like stretch stature), comparisons between groups were made using Student’s *t*-test.

For female hormone levels, which were measured at multiple time points, the researchers used a one-way analysis of variance (ANOVA) to identify any differences across those time points. Most other variables were analyzed using a two-way ANOVA, which explores the impact of two factors: sex (male or female) and measurement timing. The Bonferroni post hoc test was used to assess the effect size for the variable being measured. Partial eta squared (ƞ^2^) was calculated as an indicator of effect size, with 0.01–0.06 constituting a small effect size, 0.06–0.14 representing moderate effects and >0.14 indicating large effects [28] in terms of Zn values. Statistical significance levels were defined at *p* < 0.05 for differences deemed significant and *p* < 0.01 considered highly significant.

## 3. Results

The data presented in Table 4 reveal significant sex-based distinctions (*p* < 0.001) across all anthropometric and body composition measurements. Additionally, there were assessment-based variations (*p* < 0.05) observed in the total values of skinfolds, fat and muscle, particularly between the first and second assessments.

The data from Table 5 show the parameters obtained in the maximum incremental test. There were differences between sexes in all parameters analyzed (*p* < 0.05). Additionally, the respiratory exchange ratio differed between assessments (*p* < 0.05).

Table 6 shows the nutritional intake of macronutrients and Zn. Dietary intake also shows variations according to sex (*p*< 0.05), with male soccer players consuming more total calories and protein over the three days of the study. No differences in Zn intake were observed.

Table 7 displays the data obtained for erythrocytes and platelets. Erythrocyte counts were further influenced by the time of evaluation, with significant differences observed between evaluations 1 vs. 2 and 1 vs. 3 (*p* < 0.05). In addition, there were sex differences in erythrocyte counts.

Table 8 reports the hormone concentrations throughout the investigation. No significant differences were observed.

Extracellular (plasma and urine) and intracellular (erythrocytes and platelets) concentrations are showed in Table 9. These values are expressed in both absolute and relative to cell count quantities.

When plasma Zn concentrations were analyzed, significant differences were observed between sexes with a large effect size (*p* ≤ 0.01). The effect of the measure had a moderate effect size.

Regarding urinary Zn concentrations, there were differences between sexes and throughout the assessments (*p* ≤ 0.01), with a large effect size. Specifically, differences were observed between assessments 1 and 3 and assessments 2 and 3 (*p* ≤ 0.01). The sex × measurement interaction presented a moderate effect size.

Regarding erythrocyte Zn concentrations, no significant differences were observed when absolute concentrations were analyzed. However, differences between sexes were observed when erythrocyte concentrations were expressed in values relative to the number of erythrocytes, with a large effect size (*p* ≤ 0.05).

Finally, regarding intraplatelet concentrations of Zn, differences were observed throughout the investigation in intraplatelet concentrations expressed in absolute values (*p* ≤ 0.01) with a large effect size, as well as the difference between sexes. The differences were found between assessments 1 and 3 and assessments 2 and 3 (*p* ≤ 0.05). Regarding intraplatelet Zn concentrations expressed in values relative to the number of platelets, significant differences were observed both between sexes and throughout the assessments with large effect sizes (*p* ≤ 0.01). The differences were found between assessments 1 and 3 and assessments 2 and 3 (*p* ≤ 0.05).

## 4. Discussion

This study aimed to analyze Zn levels in various biological compartments (plasma, erythrocytes, platelets and urine) of football players at different times during the sporting season as well as to analyze Zn differences between sexes.

The results showed that male football players had higher extracellular concentrations (in plasma and urine), while female football players had higher intracellular concentrations (in erythrocytes and platelets). 

Regarding sex differences, there is no agreement in the literature in terms of Zn status yet. Studies have evaluated this mineral in different biomarkers: erythrocytes, plasma, urine and serum [29], which hampers the comparability between studies [30].

Factors such as age, hormones, stress, infections and inflammation could affect serum concentrations even though serum Zn concentration is the most widely used biomarker to determine Zn status [5,31]. Thus, other compartments need to be considered to assess Zn status. Scientific studies on intracellular zinc concentrations are limited [32]. Intracellular assessments such as that of erythrocytes could be more reliable because they appear to be unaffected by short-term diet or inflammatory processes [33]. Cytokines produced during the post-exercise inflammatory response increase capillary permeability, allowing albumin and other proteins to be redistributed into the interstitium. Zinc concentrations are bound to albumin and may therefore decrease when albumin concentrations fall [33]. Therefore, when evaluating Zn, it is important to simultaneously determine intracellular and extracellular concentrations in order to understand the kinetics of the mineral. It should be noted that all levels were within normal concentrations [33,34].

It is also important to note that the redistribution of zinc between compartments and organs may be affected by physical training [7]. Environmental temperature, type of exercise and training status are factors that affect this redistribution [8]. Micronutrients are necessary for several metabolic processes in the body and are important for supporting growth and development [35]. In the present investigation, both groups ingested Zn above the dietary reference intakes (9.5 mg/day) [7]. Książek et al. [36] showed higher Zn intakes in Polish players during the preseason compared to the present study. In Polish women players, the intake was lower than those reported in the present investigation [37]. An adequate intake of Zn in the athlete’s diet is necessary given its involvement in energy metabolism [14].

Significant changes in urinary and platelet concentrations of Zn were observed when analyzing the differences throughout the assessments. In relation to urinary concentrations, an increase followed by a decrease was observed in the male soccer players, while in the women, an inverse evolution was observed. Urinary Zn concentrations could increase due to strenuous exercise [38], regular training [22], prolonged heat exposure [11] and Zn supplementation [39]. In soccer players, a significant increase in urinary Zn levels was observed after an indoor soccer match compared to pre-match values [40,41]. Intense aerobic exercise and muscle strength training reduce zinc levels in serum and increase its elimination through urine, particularly when the exercise is performed to the point of exhaustion [21,31,42]. These modifications in Zn levels have been reported during the recovery stage, indicating that it could be related to the muscle regeneration processes undergone during this period.

Regular physical exercise can increase urinary Zn excretion via urine due to an increase in muscle protein catabolism caused by the increased load of amino acids filtered by the kidneys [43,44]. The Zn complexes produced could likely be Zn amino acid chelates that would increase the fraction of loose Zn in plasma and lead to increased renal filtration and urinary Zn excretion [45]. Similarly, increases in urinary Zn may also occur due to muscle damage [40]. The findings of Granell et al. [46] reported that the type of exercise triggers changes in serum and urinary Zn, being aggravated by muscle damage. The urinary decreases reported in both groups have been observed in other previous studies [39,47]. The decreased urinary elimination of ZN could correspond to an adaptive mechanism to avoid loss of these elements. The lower urinary elimination of Zn could correspond to an adaptive mechanism to avoid element losses [48,49].

Regarding intraplatelet Zn differences, progressive decreases were observed throughout the study. Few studies have investigated platelet Zn concentrations in athletes. One study reported that subjects with low levels of physical activity showed higher intraplatelet Zn compared to soccer players [17]. Prior research has reported that platelets could release Zn into serum by breaking down in the coagulation process [50], which could have special relevance in soccer due to the number of injuries that occur during training and competition. In addition, the aforementioned research suggested that a slight increase in plasma dilution resulting from hemolysis and platelets are possible factors in the increase in Zn concentration [50].

Regarding sex differences, male soccer players showed higher Zn concentrations in plasma and urine. However, female soccer players exhibited higher Zn concentrations in erythrocytes and platelets in relative values. 

Concerning the differences between the men and women, extracellular Zn levels were higher in the men with respect to the women, while in the women players there were higher intracellular concentrations (platelets and erythrocytes). A previous study also reported higher serum Zn levels in men after a physical test [18]. Alternatively, another study [19] found that men experienced significantly greater zinc losses through sweat compared to women under both hyperthermic and normothermic conditions. Conversely, other researchers did not detect any sex differences in zinc loss [51].

The variations in Zn levels between sexes in the different compartments examined might be attributed to several factors. Muscle mass could be a factor for the observed differences between sexes. In addition, Zn is harbored mostly in skeletal muscle [1]. Another factor could be actions such as accelerations, changes of direction and decelerations that can cause muscle damage, as well as trauma due to blows and impacts. [52,53]. The physical demands and intensity of matches are higher in men than in women [54], resulting in higher-impact collisions and consequently greater hemolysis. It is known that this phenomenon leads to increases in plasma Zn levels [33,55] because erythrocyte Zn levels are significantly higher than serum Zn levels [56]. One reason for this is that Zn is part of the membrane and is a cofactor for SOD and carbonic anhydrase [57]. Thus, the differences may be due to a greater release into the plasma from the muscle [58,59], in addition to an increase in urinary Zn. A prior study reported a fall in erythrocyte levels with a rise in plasma, such as found in the current research [59].

The variations between sexes cannot be explained by the increased Zn needs in men, as their growth rate and muscle mass per kilogram of body weight exceed those of women. Zinc is vital in the processes involved in nitrogen absorption and retention, necessary for building lean body mass and the structures that constitute the organism [57].

It should be noted that it is important to express intracellular concentrations of mineral elements in values relative to cells, since, as in the present study, the results could be different. Regarding platelets, previously in a similar study it has been reported that men show higher Zn concentrations compared to women players [55]. However, there is scant evidence to justify these differences between sexes. It could be hypothesized that men, having a greater anaerobic capacity, have a greater flow of Zn from the erythrocyte to the plasma, while in women, who are predominantly aerobic [56], the flow is greater towards the erythrocyte. In addition, hormones may be another factor to consider, since increased testosterone post-exercise has been associated with increased serum Zn. Studies have already reported that testosterone levels are higher in men than in women [60,61].

This research is not exempt from possible limitations that should be considered: the hydric levels in the organism could vary due to the physical exercise itself, influencing the plasma volume. Other complementary values such as enzymes were not measured. A sample calculation was not performed before the experimental phase.

## 5. Conclusions

Zn concentrations varied between sexes and over the season in soccer players. Specifically, male soccer players showed higher extracellular (plasma and urine) concentrations while female soccer players showed higher Zn concentrations in relation to the number of erythrocytes and platelets. Moreover, urinary and platelet concentrations decreased at the end of the season.

Muscle damage caused by physical training could lead to an increase in plasma Zn released by muscle cells. The results obtained in erythrocytes could be due to possible nutritional deficits caused in previous months or to hemolysis produced by physical training, which could also increase plasma Zn concentrations.

A multicompartmental evaluation of Zn status is suggested due to discrepancies in the evaluation of isolated extracellular and intracellular Zn compartments.

## Figures and Tables

**Table 1:** Training performed and injuries sustained during the sports season.

	Male Soccer Players	Female Soccer Players
Total training (n)	128.27 ± 18.59	133.54 ± 25.86
Total training (min)	11,814.23 ± 1673.4	10,578.46 ± 3227.80
Injuries (n)	7	8
Absence from training (days)	12.07 ± 9.34	14.14 ± 10.79

**Table 2:** Characteristics of the menstrual cycle of the female soccer players.

		Female Soccer Players
Age at menarche (years)		13.5 ± 1.15
Regular menses (%)		100.00
Duration of bleeding (days)		4.77 ± 1.47
Menstrual cycle (days)		27.93 ± 2.78
Cessation of menses (%)	Never	88.88
	Occasionally	12.22

**Table 3:** Limits of detection and limits of quantification for Zn.

Matrix	Limits of Detection (µg/L)	Limits of Quantification (µg/L)
Plasma	0.056	0.56
Urine	0.019	0.19
Erythrocytes	0.034	0.34
Platelets	0.157	1.57

**Table 4:** Anthropometric and body composition characteristics of the participants.

		Male Soccer Players	Female Soccer Players	Sex Effect	Time Effect	Sex × Time
Height (m)	1st assessment	1.76 ± 0.061	1.65 ± 0.06 ++	-	-	-
	-	-	-			
	-	-	-			
Weight (kg)	1st assessment	71.50 ± 5.93	59.58 ± 7.17	<0.001	0.748	0.931
	2nd assessment	71.95 ± 5.87	60.44 ± 6.77			
	3rd assessment	72.80 ± 5.68	66.39 ± 8.99			
Σ6 Skinfold (mm)	1st assessment	60.34 ± 12.35	94.62 ± 18.54	<0.001	0.009	0.016
	2nd assessment	60.12 ± 12.61	76.72 ± 15.13 *			
	3rd assessment	56.85 ± 12.12	83.81 ± 18.75			

* *p* ≤ 0.05 differences between 1st and 2nd assessment; ++ *p* ≤ 0.01 differences between 1st and 3rd assessment.

**Table 5:** Results obtained from the incremental treadmill test throughout the study.

		Male Soccer Players	Female Soccer Players	Sex Effect	Time Effect	Sex × Time
Time (min)	1st assessment	12.41 ± 1.58	9.18 ± 1.12	<0.001	0.117	0.345
	2nd assessment	12.38 ± 1.48	8.57 ± 1.21			
	3rd assessment	12.16 ± 1.89	8.11 ± 1.64			
Maximum speed (km/h)	1st assessment	19.17 ± 1.72	15.73 ± 1.16	<0.001	0.289	0.315
	2nd assessment	19.22 ± 1.44	15.20 ± 1.10			
	3rd assessment	19.15 ± 1.98	14.91 ± 1.37			
VO_2max_ (L/min)	1st assessment	2.10 ± 0.20	1.40 ± 0.25	<0.001	0.337	0.296
	2nd assessment	2.15 ± 0.22	1.48 ± 0.18			
	3rd assessment	2.00 ± 0.29	1.48 ± 0.17			
RER	1st assessment	1.12 ± 0.03	1.16 ± 0.04	0.042	<0.001	0.017
	2nd assessment	1.07 ± 0.02 **	1.08 ± 0.02 **			
	3rd assessment	1.08 ± 0.02 ++	1.09 ± 0.03 ++			
HR (bpm)	1st assessment	187.78 ± 6.52	183.33 ± 7.34	<0.001	0.177	0.204
	2nd assessment	188.90 ± 5.82	179.75 ± 8.11			
	3rd assessment	186.90 ± 7.42	176.90 ± 8.00			
Oxygen pulse (mL/beat)	1st assessment	19.44 ± 1.85	12.48 ± 2.24	<0.001	0.109	0.287
	2nd assessment	20.08 ± 2.34	13.80 ± 1.63			
	3rd assessment	19.21 ± 2.221	13.90 ± 1.66			
VE (L/min)	1st assessment	134.73 ± 13.75	81.40 ± 15.89	<0.001	0.304	0.542
	2nd assessment	136.04 ± 17.07	89.90 ± 12.06			
	3rd assessment	134.65 ± 15.93	83.40 ± 11.36			

** *p* ≤ 0.01 differences between 1st and 2nd assessment; ++ *p* ≤ 0.01 differences between 1st and 3rd assessment; VO_2max_: maximal oxygen uptake; RER: respiratory exchange ratio; HR: heart rate; VE: expired volume.

**Table 6:** Mean intake of the three days of evaluation of macronutrients and Zn throughout the study.

		Male Soccer Players	Female Soccer Players	Sex Effect	Time Effect	Sex × Time
Energy (kcal)	1st assessment	1796.0 ± 420.0	1578.1 ± 316.2	0.038	0.497	0.317
	2nd assessment	1932.2 ± 312.5	1681.5 ± 427.3			
	3rd assessment	1882.7 ± 358.6	1697.3 ± 386.1			
Proteins (g)	1st assessment	106.1 ± 25.5	90.4 ± 21.6	0.047	0.469	0.218
	2nd assessment	115.5 ± 23.4	96.2 ± 18.3			
	3rd assessment	108.9 ± 24.8	92.6 ± 20.4			
Lipids (g)	1st assessment	54.8 ± 19.1	48.3 ± 12.3	0.116	0.241	0.471
	2nd assessment	64.1 ± 15.4	55.6 ± 15.3			
	3rd assessment	58.6 ± 17.4	60.3 ± 20.6			
Carbohydrates (g)	1st assessment	231.0 ± 69.1	206.1 ± 81.3	0.471	0.856	0.683
	2nd assessment	235.8 ± 60.3	241.5 ± 56.1			
	3rd assessment	242.0 ± 57.0	235.8 ± 61.7			
Zn (mg)	1st assessment	10.7 ± 3.1	9.3 ± 2.5	0.367	0.732	0.581
	2nd assessment	11.4 ± 2.4	10.1 ± 3.4			
	3rd assessment	11.1 ± 2.9	10.5 ± 2.7			

Zn: zinc.

**Table 7:** Erythrocyte and platelet values according to sex throughout the study.

		**Male Soccer Players**	**Female Soccer Players**	**Sex Effect**	**Time Effect**	**Sex × Time**
Erythrocytes (millions)	1st assessment	4.92 ± 0.36	4.37 ± 0.22	<0.001	0.031	0.063
	2nd assessment	4.83 ± 0.32 **	4.19 ± 0.27 **			
	3rd assessment	4.99 ± 0.29 ++	4.35 ± 0.27 ++			
Platelets (thousands)	1st assessment	204.50 ± 57.65	196.00 ± 38.01	0.274	0.542	0.222
	2nd assessment	196.60 ± 39.79	219.08 ± 34.19			
	3rd assessment	195.13 ± 37.82	204.39 ± 31.52			

** *p* ≤ 0.01 differences between 1st and 2nd assessment; ++ *p* ≤ 0.01 differences between 1st and 3rd assessment.

**Table 8:** Hormones present in females during the course of a sports season.

		Female Soccer Players	*p*
Progesterone (ng/mL)	1st assessment	2.65 ± 3.88	0.998
	2nd assessment	2.38 ± 3.21	
	3rd assessment	2.31 ± 2.89	
Estradiol-17beta (pg/mL)	1st assessment	74.04 ± 45.30	0.894
	2nd assessment	71.32 ± 39.25	
	3rd assessment	68.30 ± 40.93	

**Table 9:** Zn concentrations in plasma, urine, erythrocytes and platelets throughout the study in the different groups.

		Male Soccer Players (CI 95%)	Female Soccer Players (CI 95%)	Sex Effect	Time Effect	Sex × Time
Plasma (µg/L)	1st assessment	956.70 ± 194.36 (897.66–1098.54)	775.15 ± 121.60 (729.45–848.36)	<0.001 #	0.124 $	0.676
	2nd assessment	998.69 ± 175.85 (890.26–1050.70)	839.28 ± 136.61 (742.47–908.80)			
	3rd assessment	994.87 ± 79.55 ^ (974.39–1142.98)	874.09 ± 80.18 ^ (854.97–950.56)			
Urine (µg/L)	1st assessment	874.48 ± 334.83 (757.02–1116.13)	734.16 ± 277.19 (634.67–865.13)	<0.001 #	0.001 #	0.058 $
	2nd assessment	1172.75 ± 413.28 ^^ (1087.97–1664.66)	300.59 ± 235.07 ^^ (238.99–430.65)			
	3rd assessment	554.54 ± 149.19 ++ (549.69–825.91)	533.80 ± 275.50 ++ (477.64–796.41)			
Absolute erythrocytes (mg/L)	1st assessment	9.80 ± 1.10 (9.00–10.30)	9.90 ± 1.89 (9.21–11.16)	0.556	0.301	0.475
	2nd assessment	10.34 ± 1.02 (10.85–12.05)	10.67 ± 1.78 (10.78–12.33)			
	3rd assessment	10.72 ± 1.14 (10.57–11.88)	10.44 ± 1.21 (9.07–11.32)			
Relative erythrocytes (µg/cell^−6^)	1st assessment	2.08 ± 0.12 (1.22–1.96)	2.37 ± 0.47 (2.19–2.67)	<0.001 #	0.218	0.740
	2nd assessment	2.29 ± 0.27 (2.23–2.60)	2.63 ± 0.48 (2.43–2.84)			
	3rd assessment	2.18 ± 0.35 (2.09–2.42)	2.42 ± 0.31 (2.06–2.61)			
Absolute platelets (µg/L)	1st assessment	270.85 ± 55.02 (196.05–271.84)	287.79 ± 48.33 (215.28–312.39)	0.087 #	<0.001 #	0.204
	2nd assessment	272.90 ± 79.39 ^^ (246.34–315.28)	187.65 ± 66.91 ** (154.91–207.71)			
	3rd assessment	204.73 ± 61.69 ++ (179.05–271.15)	184.53 ± 58.54 ++ (152.96–227.82)			
Relative platelets (pg/cell^−3^)	1st assessment	1.53 ± 0.50 (0.82–1.64)	1.45 ± 0.26 (1.11–1.68)	0.002 #	<0.001 #	0.163
	2nd assessment	1.35 ± 0.25 ^^ (1.28–1.60)	0.855 ± 0.269 ** (0.71–0.91)			
	3rd assessment	1.02 ± 0.24 ++ (0.90–1.47)	0.919 ± 0.360 ++ (0.74–1.15)			

#: large effect size (>0.14); $: moderate effect size (0.06–0.14); ** *p* ≤ 0.01 differences between 1st and 2nd assessment; ++ *p* ≤ 0.01 differences between 1st and 3rd assessment; ^ *p* ≤ 0.05 differences between 2nd and 3rd assessment; ^^ *p* ≤ 0.01 differences between 2nd and 3rd assessment; Zn: zinc.

## Data Availability

The original contributions presented in the study are included in the article, further inquiries can be directed to the corresponding authors.
